# A qualitative exploration of changes and mechanisms of changes in a psychoeducational intervention for family dementia caregivers

**DOI:** 10.1186/s12875-024-02602-2

**Published:** 2024-09-28

**Authors:** Stephanie Kipfer, Cedric Mabire, Jean Vézina, Andrea Koppitz, Sandrine Pihet

**Affiliations:** 1 HES-SO University of Applied Sciences and Arts Western Switzerland – Fribourg, School of Health Sciences, Fribourg, Switzerland; 2 Institute of Higher Education and Research in Healthcare-IUFRS, University of Lausanne, Lausanne University Hospital, Lausanne, Switzerland; 3 School of Psychology, University Laval, Québec City, Canada

**Keywords:** Dementia, Family caregivers, Psychoeducational intervention, Complex interventions, Change mechanism, Qualitative research, Grounded theory, Nursing

## Abstract

**Background:**

‘Learning to feel better… and help better’ is a psychoeducational intervention that aims to empower family caregivers of people with dementia by helping them cope better with the daily stress of dementia caregiving. The intervention has been adapted to a Swiss context and evaluated with a mixed-method design, yielding promising results in caregivers, such as a reduced subjective burden and improved self-efficacy. Qualitative findings have provided insight into potentially relevant intermediate changes that must be further explored to better understand how the intervention precipitates the achieved changes. We aim to qualitatively explore such changes, related mechanisms and key intervention components in the context of this intervention.

**Methods:**

A constructivist grounded theory approach was used to achieve this aim. Changes, related mechanisms and key intervention components were identified by exploring the following: 1) longitudinal qualitative data, collected from 13 family caregivers via interviews performed before, during and after the intervention (39 interviews total) and 2) cross-sectional post-intervention interview data collected from 22 family caregivers (22 interviews).

**Results:**

Experiencing calmness was the most important change for caregivers in the context of this intervention. The calmness model, developed based on the qualitative analysis, illustrates the intermediate changes that contributed to calmness, such as being able to cope with daily life and experiencing positive interactions with the family member with dementia. Related key intervention components were the coping strategy ‘reframing’, employed in diverse ways by the caregivers to reduce daily stress, and the didactic method ‘active skills’ training’, which involved active participation by the caregivers and the guidance of a professional group leader. One important factor hampering changes in caregivers was having difficulties accepting the caregiver role or accepting the losses due to dementia.

**Conclusion:**

The calmness model offers valuable insight into how this intervention can benefit family caregivers and aid in developing interventions targeting similar mechanisms and changes.

**Trial registration:**

ISRCTN13512408 (registration date 17.05.2021, retrospectively registered).

**Supplementary Information:**

The online version contains supplementary material available at 10.1186/s12875-024-02602-2.

## Background

Family caregivers of individuals living with dementia provide regular support to enable them to remain in their own homes within their communities. These caregivers play a crucial role in facilitating their daily life management and in helping them maintain their sense of personhood [1–4]. However, caring for a person living with dementia can be associated with chronic stress and a high subjective burden, leading to physical, psychological, emotional, social and financial problems [3, 5–8]. Family caregivers must cope with losses and changes in the behaviour of their relative with dementia as well as in their relationship with them [9, 10]. These caregivers also have to assume new responsibilities, acquire new skills and plan for the future [9, 11]. However, family caregivers often have no experience in providing care, frequently feeling unprepared and lacking the required competencies to deliver appropriate care [11, 12]. To reduce negative outcomes, caregivers need support which is tailored to their needs and which helps them to effectively adopt their role, manage and maintain the caregiving situation, and sustain their relationship with their family member living with dementia [13].

‘Learning to feel better… and help better’ (LFBHB) is a psychoeducational intervention which aims to empower family dementia caregivers to better cope with the daily stress of dementia caregiving, including the management of dementia-related behaviours. It was originally developed and evaluated with a randomised controlled trial (RCT) and a process evaluation in Quebec, Canada [14–16]. The RCT revealed a reduced frequency of behaviour problems in people with dementia and a decrease in caregivers’ stress reactions to these behaviours compared to control participants of support groups [14]. Since 2015, the LFBHB intervention has been adapted and evaluated in Switzerland in several consecutive steps, including two feasibility and pilot trials with mixed-methods one-group designs. The first feasibility and pilot study indicated substantial and significant improvements in the caregivers’ subjective burden, psychological distress and self-efficacy. However, the recruitment of the participants was challenging [17]. Therefore, a research team adapted the LFBHB intervention to facilitate participation, using a participatory approach. The number of sessions was reduced from 15 two-hour sessions to seven three-hour sessions. Short educational videos were introduced to condense and standardise the content of the intervention [18]. With the shortened LFBHB intervention, it was possible to include more participants not yet in contact with health care professionals, compared with the intervention’s longer version. However, the results were similar to those associated with the longer version, namely, significant improvements in subjective burden, psychological distress and stress reactions of family caregivers in response to the behaviour problems of the persons living with dementia [18].

Studies in Switzerland [17, 18] and Canada [14] have yielded promising results regarding family caregivers. However, according to the Medical Research Council (MRC) framework, developing and evaluating complex interventions requires not only knowing whether the desired changes in the outcomes can be achieved, but also understanding ‘how an intervention is expected to lead to its effects and under what conditions’ [19, p.4]. This understanding should be described in the programme theory, which should ideally be developed at the beginning of a research project and refined during the subsequent phases [19]. Understanding how changes develop, through which mechanisms and key intervention components, and which contextual aspects are facilitating or preventing these changes is essential to refine the programme theory and optimise the intervention [19–22].

The qualitative analyses performed in the first and second feasibility and pilot studies in Switzerland provided insight into relevant aspects which warranted further exploration to better understand the outcomes and the mechanisms of change in the LFBHB intervention. Exploring these aspects allows to refine the intervention’s theoretical basis and optimise the intervention itself. The qualitative analyses indicated that the relationship quality was particularly relevant. This finding is consistent with the theoretical and empirical literature describing relationship quality as an important determinant of the well-being of both people in the caregiving dyad [23]. Consequently, a longitudinal qualitative constructivist grounded theory study was performed to explore relationship quality in the LFBHB intervention [24, 25]. This study resulted in the **S**ustaining **R**elationship **Q**uality in **D**ementia (SRQD) model, which illustrates the supportive strategies caregivers applied to sustain or maintain relationship quality as well as the knowledge and skills they required to master such strategies. It further describes the key components of the LFBHB intervention regarding relationship quality and aspects which facilitated or prevented caregivers from developing and applying supportive strategies [24].

The qualitative data on relationship quality indicated that there might be bidirectional links between relationship quality and other changes experienced by the caregivers. To achieve a more complete understanding of the intervention’s change process, more knowledge was needed about the nature of these other changes, occurring in addition to those in relationship quality (hereafter, additional changes), their associated change mechanisms as well as their interactions with each other. The process evaluation for the LFBHB intervention conducted in Canada [16] highlighted the educational and group support processes related to the outcomes used in their RCT. However, having applied the LFBHB in a different cultural context and having shortened and adapted it to facilitate participation, a new exploration of the change processes and mechanisms was needed to refine the programme theory. Therefore, a qualitative data analysis was performed to explore the additional changes and the related change processes in the context of the Swiss shortened LFBHB intervention used in Switzerland. More specifically, the aim was to qualitatively explore the following: 1) the additional changes in the caregivers during and after their participation in the intervention (aim 1); 2) the mechanisms contributing to such changes, including the key intervention components involved (aim 2); 3) the contextual elements facilitating or preventing these changes in caregivers (aim 3).

## Methods

### Design

An interpretative constructivist grounded theory approach [26] was used to explore additional changes and related processes in the context of the Swiss shortened LFBHB intervention. Change processes were identified in two phases. The first phase explored qualitative longitudinal data about the change process in relationship quality as perceived by the caregivers over time (described in Kipfer et al. [24, 25]). Data were collected amongst caregivers before, during and after their participation in the LFBHB intervention. This led to the SRQD model [24]. The second phase is a complementary qualitative exploration of cross-sectional data which is presented in the current publication. The reporting of this study was guided by the consolidated criteria for reporting qualitative research (COREQ) [27].

### The ‘Learning to feel better… and help better’ intervention

‘Learning to feel better… and help better’ (LFBHB) is a psychoeducational group intervention for informal dementia caregivers who care for community-dwelling persons living with dementia. It aims to help family caregivers to better cope with the daily stress of dementia caregiving. The content is based on the transactional theory of stress and coping proposed by Lazarus and Folkman [28, 29]. The intervention focuses on a systematic procedure guiding the appraisal of stressful situations and the application of appropriate coping strategies. Coping strategies applied in the intervention include problem-solving, the reframing of unhelpful thoughts and support seeking. In addition, caregivers receive information regarding the impact of the disease on the communication and behaviour of the person living with dementia. They also receive information about appropriate communication and caring approaches to prevent tension and stressful situations in daily life. The intervention combines information provision, group discussions and active skills training to apply the systematic procedure to personal situations. It further incorporates exercises at home to facilitate knowledge transfer. Additional File 1 provides a summary of the content and didactic tools. The intervention is guided by trained health care professionals. In the Swiss version used in this study, the intervention was led by a psychologist and/or a nurse. The Swiss version of the intervention is delivered in person to small groups of 4 to 8 caregivers, over 6 weekly sessions lasting 3 hours each, in addition to a follow-up session one month after the last weekly session.

### Recruitment process

Information about the LFBHB intervention was shared through articles in local newspapers and by professionals and organisations supporting family caregivers. Family caregivers willing to participate and fulfilling the following eligibility criteria were invited to take part in the study: 1) regularly providing unpaid care to a person living in the community and having a diagnosis of dementia or exhibiting substantial cognitive deficits; 2) being 18 years or older; and 3) having sufficient language skills in German.

### Ethical considerations

The study was approved by the local Swiss ethics review board (Commission cantonale (Vaud) d'éthique de la recherche sur l'être humain, protocol n°175/14, ISRCTN13512408) and performed according to the Helsinki Declaration [30]. A member of the research team informed each family caregiver interested in participating in the study about the study aims, the data collection procedure, data protection and confidentially, as well as their rights as participants and potential benefits or inconveniences when participating in the study. Written and oral informed consent was obtained from each family caregiver before data collection.

### Data collection

Data was collected in five different LFBHB groups conducted with German-speaking participants between September 2020 and November 2022. Data collection involved two phases. In the first phase, qualitative longitudinal data focusing on relationship quality was collected in three semi-structured interviews performed before (t0), during (t1) and after (t2) the intervention. These interviews were performed in the first three consecutive groups (N=13), resulting in 39 interviews. The interview guide for this first phase is presented in Kipfer et al. [24]. These longitudinal data provided essential information about the mechanisms behind changes in relationship quality and about additional changes observed in the caregivers. It further indicated that these different changes and the related mechanisms interacted with each other. To deepen the understanding around these additional changes, a second analysis was performed on the qualitative post-intervention interviews evaluating the LFBHB intervention in five LFBHB groups (N=22). These interviews aimed at evaluating the benefits and negative aspects of the LFBHB intervention as well as the experiences of the caregivers in relation to the intervention. They were performed within one to three weeks after the end of the intervention. The interviews took place at participants’ homes, at the university or by phone according to the participants’ preferences and accounting for applicable COVID sanitary measures. Only the participants and the interviewers were present during these interviews. The interviews of the first three groups were conducted by the first author (SK), a nurse who is experienced in conducting qualitative research and working with family caregivers. Interviews of groups four and five were performed by a female research collaborator with a master’s degree in psychology or one of two female research assistants, all of whom trained in performing qualitative interviews. The interviewers were not involved in providing the intervention and had no relationship to the participants. Before the beginning of the interviews, participants were informed about the interviewers’ professional backgrounds. The interviews followed a semi-structured interview guide [see Additional File 2] that was developed and pilot tested in a prior pilot study evaluating the LFBHB intervention [18]. The semi-structured format allowed to adapt questions or add questions to explore new emerging themes. The open-ended questions focused primarily on how caregivers experienced their participation in the intervention. Further questions explored intervention aspects perceived as negative or positive by the caregivers or possibilities to improve the intervention. Fieldnotes were taken during the interviews to record relevant observations or information. The interviews lasted 13 to 69 minutes, with a mean time of 36 minutes. They were audio-recorded, pseudonymised and verbally transcribed. To minimise participants’ efforts, transcripts were not submitted to them for comments unless they requested it, which none of them did. Table 1 provides an overview of the data collected in the five LFBHB groups.

**Table 1:** Data analysed from the five LFBHB intervention groups

**Group**	**Intervention time**	**Nb. participants**	**Nb. spouse CG**	**Nb. adult child CG**	**t0**			**t1**	**t2**		
					**Demogr. data**	**Interview RQ**	**Quanti. data**	**Interview RQ**	**Interview RQ**	**Quanti. data**	**Evaluation interview**
**1**	Sep–Nov 2020^a^	5	4	1	**x**	**x**	**x**	**x**	**x**	**x**	**x**
**2**	Mar–May 2021^b^	4	4		**x**	**x**	**x**	**x**	**x**	**x**	**x**
**3**	Apr–Jun 2021^b^	4	2	2^c^	**x**	**x**	**x**	**x**	**x**	**x**	**x**
**4**	Oct–Dec 2021	5	5		**x**		**x**			**x**	**x**
**5**	Sep–Nov 2022	4	2	2	**x**		**x**			**x**	**x**
**Total**		**22**	**17**	**5**	**22**	**13**	**22**	**13**	**13**	**22**	**22**

^a^During second wave of COVID-19 pandemic, thus with restricted group size

^b^During third wave of COVID-19 pandemic, thus with restricted group size

^c^Including one child-in-law

*CG* Caregiver, *RQ* Relationship quality

Quantitative data were collected regarding the 22 participants to evaluate the effects of the LFBHB intervention. These data were collected within two weeks prior to the start of the intervention and within two weeks post intervention. Quantitative data were collected to assess changes in caregivers’ subjective burden, psychological distress and self-efficacy, as well as in the memory and behavioural problems (MBP) of the persons living with dementia and associated distress in the caregivers. Table 2 and Additional File 3 delineate these measures. The demographic and health data of the caregivers and persons living with dementia were collected at pre-test. The individual demographic and quantitative data of each participant were considered as additional information in the qualitative analysis to explore similarities and differences between the caregivers regarding achieved changes and related mechanisms, as well as other potentially relevant factors.

**Table 2:** Quantitative outcomes of the LFBHB intervention

**Quantitative outcome**	**Measurement instrument**	**Items and response scale**
Caregiver burden	Zarit Burden Interview [31, 32]	22 items, response scales ranging from 0 (never) to 4 (very often); total scores > 18 indicated a heavy burden and, > 32 indicated severe burden
MBPs and caregivers’ MBP-related distress	Revised Memory and Behavior Problems Checklist (RMBPC) [33]	Frequency of 24 MBPs in the preceding week, scored from 0 (never) to 4 (daily), and the extent to which each MBP disturbed or upset the caregiver, scored from 0 (not at all) to 4 (extremely)
Caregivers’ psychological distress	Ilfeld Psychiatric Symptoms Index – Short Version [34]	Rating of 14 symptoms related to depression, anxiety, anger and cognitive disturbance in the preceding week, a 4-point scale from 1 (never) to 4 (very often)
Caregivers’ self-efficacy	Bandura [35]	1 item about confidence regarding the ability to assume the caregiver role that uses a visual analogue scale ranging from 0 (no confidence at all) to 10 (full confidence)

*LFBHB* Learning to feel better… and help better, *MBP* Memory and behavioural problems

### Qualitative data analysis

All 22 post-intervention evaluation interviews of phase two were first analysed with a focus on caregivers’ experiences while participating in the LFBHB intervention. A coding system reflecting this focus was developed. The data analysis started after the first interview and was then performed in parallel with the subsequent interviews to evaluate the benefits and negative aspects of the shortened LFBHB intervention as well as the experiences of the caregivers participating in the intervention. To deepen the understanding regarding the interactions of relationship quality with other qualitative findings and change mechanisms in the LFBHB intervention, all 22 post-intervention evaluation interviews were then coded a second time with the coding system developed in the first phase focusing on relationship quality. The two coding systems were then combined to identify shared themes and possible interacting elements. Data analyses for both phases followed the procedure of Charmaz [26] with initial, focused and theoretical coding. Constant comparison was used in all steps to develop more abstract constructs regarding the phenomena explored in the analysis. Memos and informal analytical notes documented the different steps of the analysis, particularly the processes and decisions made when developing the coding system and the two theoretical models.

All interviews were coded by the first author (SK) and assessed by a second coder (SP) who coded a random sample of excerpts and interviews with the developed coding system. Differences in coding, definitions of codes as well as findings and properties were regularly discussed between the two coders as well as with other researchers and clinicians working with family dementia caregivers. This was important to increase the findings’ reliability and credibility and to reduce errors of interpretation [36]. The findings and the resulting model – the calmness model – were discussed in two group interviews with a total of five former participants in the LFBHB intervention. The five participating caregivers included two female spouses, one female adult child and two male spouses. These caregivers discussed and assessed whether the findings and the calmness model resonate with their experiences regarding their participation in the intervention [26]. Two similar interviews were performed with two psychologists involved in providing the intervention, to assess consistencies with their experiences when leading the LFBHB group and supporting family caregivers.

### Quantitative data analysis

Descriptive statistics were used to describe and analyse the data related to study outcomes at pre- and post-test (median, Q1 and Q3, as some distributions deviated from normality). Changes in each of the five outcomes between pre- and post-intervention were tested with non-parametric Wilcoxon tests (α = .05) computed with the SPSS software, and the effect sizes were calculated (Cohen’s d).

### Sample characteristics

Table 3 provides an overview of the sample characteristics. The 22 family caregivers participating in the study were mainly women (77%) and spouse caregivers (77%) with a mean age of 67.4 years. Most of the family caregivers (82%, i.e. all spouse caregivers and one adult child caregiver) lived in the same household as the person living with dementia. They exhibited a large variability in the time they had been providing care to their relative, varying between 6 months and 12 years. Two thirds of the people with dementia were male. Alzheimer’s disease was the most common type of dementia (41%), followed by non-specific dementia diagnoses (36%). Regarding the intervention, the caregivers participated in 96% of the seven sessions on average, with 17 of the 22 caregivers participating in all sessions. Missed sessions were mainly due to caregivers being on holiday (four sessions) or an emergency in the family (two sessions). Across all five groups, three participants were excluded from the analysis. One adult child caregiver dropped out after session four, due to the institutionalisation of the person living with dementia. A spouse caregiver decided to cease their participation after the first session, feeling it was too early as their partner had received the dementia diagnosis very recently and was still very autonomous. Another spouse caregiver declined to participate in the research project and took part in the intervention without providing data.

**Table 3:** Characteristics of the participating family caregivers and the persons they supported (n=22)

**Characteristics of the family caregivers (n=22)**		
Age	Mean = 67.40 years (Range: 44 – 84 years)	
Gender	Female Male	77% (n = 17) 23% (n = 5)
Relationship type	Spouse caregiver Adult child caregiver Adult child in-law caregiver	77% (n = 17) 18% (n = 4) 5% (n = 1)
Housing situation	Living in same household Living in different households	82 % (n = 18) 18 % (n = 4)
Duration providing care	Mean = 3.04 years (Range: 6 months – 12 years)	
Time per week spent caregiving	Mean = 5.72 days (Range: 0.5 – 7 days)	
**Characteristics of the persons living with dementia (n=22)**		
Age	Mean = 77.45 years (Range: 58 – 92 years)	
Gender	Female Male	36% (n = 8) 64% (n = 14)
Diagnosis	Alzheimer’s disease Frontotemporal dementia Dementia with Lewy bodies Vascular dementia No specific dementia diagnosis	41% (n = 9) 9% (n = 2) 5% (n = 1) 9% (n=2) 36% (n = 8)
Duration of illness	Mean = 3.50 years (Range: 6 months – 12 years)	

## Findings

**Fig. 1:**
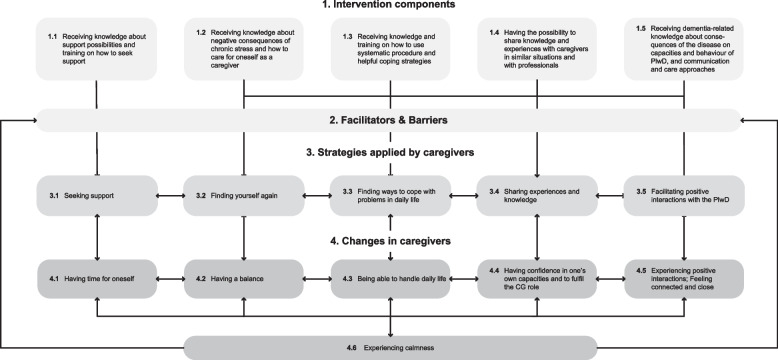
Overview of the calmness model

The first and second phases of the analysis revealed ‘experiencing calmness’ as the main change described by the caregivers participating in the Swiss short version of the LFBHB intervention. Based on these analyses, a model about experiencing calmness (hereafter named the calmness model) was developed. Figure 1 provides an overview of the different components of the model. The calmness model describes the main change (i.e. experiencing calmness) as well as associated changes that the caregivers experienced during and after their participation in the LFBHB intervention (level 4 in the figure, aim 1). It further depicts the mechanisms of change, including the strategies that caregivers used to facilitate these changes (level 3) as well as the intervention components (level 1) that helped them develop and apply these strategies (aim 2). Contextual aspects related to caregiver factors that facilitated or prevented the caregivers’ learning processes are illustrated as ‘Facilitators and Barriers’ (level 2, aim 3). The calmness model complements the SRQD model, which presents the changes and strategies regarding relationship quality described by caregivers in the context of the LFBHB intervention [24].

## Changes in caregivers (level 4 in Fig. 1)

### Main change: Experiencing calmness (4.6)

Experiencing calmness was mentioned by most caregivers as the most important change. Calmness was mainly described as perceiving oneself as more serene; less tense, nervous or angry; more patient; and finding it easier to handle the caregiving situation. Some caregivers reported that experiencing calmness helped them find their humour and happiness again. Caregivers reported being able to remain calmer; being kinder and less dismissive of the person living with dementia in daily caregiving situations; and responding with less stress and irritation in challenging situations. They also described perceiving their family member with dementia as calmer and less stressed in interactions with the caregiver or in daily life in general.

*‘I am calmer. I can control it better. I am less irritated if something goes wrong. I tell myself, this is the disease. That helps right away. […] He [partner with dementia] became calmer through this. […] And of course, I am gentler with him. I try to see it differently from before. I am calmer and through this I can accept him better’ (Spouse caregiver (CG) 3, t1).*

Experiencing enrichment and accepting changes were described by caregivers as being associated with experiencing calmness. Enrichment was perceived in situations wherein caregivers were able to help the family member with dementia and avoid challenging situations, when their time spent together satisfied both dyad members, or when the relatives with dementia could engage in meaningful activities. These enriching experiences further encouraged and confirmed caregivers in their caregiving behaviour and their caregiver role.

*‘I feel more competent. It makes me happy, when I see that I can really help’ (Adult child CG5, t2)*.

Accepting changes implied accepting the situation of progressive decline and not knowing how and when a decline will occur, accepting situations in which caregivers cannot help the person living with dementia and the need to accept support from other people. It also meant acknowledging their negative emotions to move forward in the acceptance process. Some caregivers reported having changed their view of their caregiver role, viewing it as voluntary rather than an imposed or burdensome duty.

‘*It is not against me. I don’t have* *to do* *everything. It still depends on me, but I do it differently. It isn’t a burden anymore. It now is a task and currently I can fulfil this task’ (Spouse CG2, t1).*

Experiencing calmness was facilitated by five associated changes and the strategies precipitating them. This relationship was found to be bidirectional, meaning that experiencing calmness in turn further strengthened the associated changes and related strategies. Calmness, for example, allowed caregivers to have a clearer head and more resources to reflect on the caregiving situations, to seek information, to be more creative in finding solutions and to execute necessary tasks.

*‘I have ideas again how to solve something. If I am calmer, I am more constructive. That is what I realised. To reflect: I could have said this differently and I can do it better next time’ (Spouse CG1, t1)*.

### Associated change: Being able to handle daily life (4.3)

Caregivers who were able to handle daily life could help themselves by knowing how to manage challenging situations and apply appropriate supportive strategies. They described two particularly facilitating aspects in this regard: 1) ‘understanding the needs and behaviours of the family member with dementia’ and 2) ‘being prepared’.

First, understanding the needs and behaviours of the family member living with dementia meant that caregivers developed a firm understanding of what it means to live with dementia and that they were able to recognise dementia-related symptoms and needs in their family member. With this knowledge, they could adapt their behaviours and the environment to the needs of the affected person to preclude negative experiences and emotions in both members of the caregiving dyad as well as to promote positive interactions.

*‘We [adult child and spouse caregiver of a person living with dementia] definitely see a progress, particularly regarding the understanding of dementia as well as the reactions and behaviour of my father. We are calmer because we can understand him better. We also know better what to do’ (Adult child CG5, t1).*

Second, being prepared implied being able to apply the systematic procedure taught in the LFBHB intervention to manage the caregiving situation and knowing what to expect in the future and who to turn to for information and support. Caregivers were prepared when they knew how to find solutions independently and therefore were not at the mercy of a situation.

*‘ […] The clear structure of possible actions allows me to feel competent and to act in critical situations. […] The guideline [systematic procedure] and the three possibilities to act [problem solving, reframing, support seeking] help me to analyse the situation. This comes really fast to my mind and thus I don’t feel helpless’ (Adult child CG5, t2).*

### Associated change: Having confidence in one’s own capacities and to fulfil the caregiver role (4.4)

When feeling confident in their capacities, caregivers dared to attempt different solutions in their daily lives and trusted that they could positively influence their own situations. This was facilitated by having knowledge about efficient strategies to cope with problems and promote positive interactions as well as by having had positive experiences when applying some of these strategies. This change further implied having the confidence to speak about their own situation and their caregiver role in the group as well as with family members or friends. This was enhanced by sharing experiences in the group with other caregivers in similar situations and a supportive health care professional.

*‘I was full of tears when I talked about it […] After that, I was able to better talk about this theme. I think I could let go of something. […]. I think it helped me to feel better and I now also have more capacities to reflect on how I can improve the situation of my husband’ (Spouse CG1, t2).*

Caregivers who exhibited less or no confidence in finding solutions often stated that they had already tried everything without success. One of these caregivers, for example, reported that they could not positively influence their situation, as they were struggling with a poor relationship with their partner, already before the onset of dementia.

### Associated change: Having time for oneself (4.1)

Having time for oneself involved being able to take breaks, to follow one’s own rhythm and to engage in activities which are important for oneself. Such respite times helped caregivers to relax and recharge their batteries, to change their minds by talking with other people and thus to have a clearer mind, remain balanced and experience calmness.

*‘He [partner with dementia] was with a friend. That day was like a present. For once, to live a day on my own rhythm and do whatever I like. I did a bike tour. […] Just something I cannot do with him’ (Spouse CG1, t2).*

Having time for oneself was mainly facilitated by the activities caregivers could engage in without support from others, such as pursuing a creative hobby at home. Being aware of the importance of caring for oneself and taking breaks encouraged caregivers to initiate, maintain or restart such activities in their daily lives. In a minority of occasions, such respite times were facilitated by the caregivers actively seeking support and receiving it from other family members or friends.

*‘I read and I carve stones. Working with stone gives me peace. […] I do a lot for myself, emotionally, to keep my balance’ (Spouse CG2, t1).*

### Associated change: Having a balance (4.2)

Having a balance was characterised by being able to relinquish inappropriate control and excessive responsibility, being able to focus on the present and being able to balance positive and negative elements. Relinquishing inappropriate control and excessive responsibility requires accepting that some situations cannot be influenced, changed or resolved. This allowed caregivers to adapt their expectations and to let matters evolve on their own; as a result, they experienced fewer negative emotions, felt calmer and enjoyed themselves more.

*‘I feel freer. I am much more carefree. I just left the house. There is a handyman coming to our house. It doesn’t matter what happens, he knows what to do. Somehow, I don’t feel so responsible anymore. I don’t think I have to control everything. I can just let it go’ (Spouse CG2, t1).*

Focusing on the present and finding a balance between positive and negative elements entailed worrying less about the future, being grateful for positive factors, enjoying small pleasures, searching for positive thoughts and not giving substantial room to negative ones, being less critical with oneself and approaching matters with humour. It further implied focusing on aspects which still worked rather than on losses or aspects negatively affected by the disease, such as the impaired capacities of the family member living with dementia.

*‘The most positive for me is […] to try to see the beautiful things, the things which remain despite the disease. To value the small things. That is what helped me the most’ (Adult child CG11, t2)*.

### Associated change: Experiencing positive interactions, feeling connected and close (4.5)

Experiencing positive interactions was characterised by caregivers receiving positive responses from the persons living with dementia, such as being motivated to participate in activities, expressing gratitude for the support of the caregiver or being calmer and displaying less distress. A better understanding of the behaviours and the needs of the family member living with dementia and an improved awareness of one’s own responsibilities and limits facilitated positive interactions within the caregiving dyad. The positive interactions allowed them to feel close and to experience increased mutuality and affection in their relationship. Sharing joyful activities further allowed caregivers to feel more connected to the family member living with dementia. Caregivers applied the following strategies (3.5) to facilitate positive interactions: letting go of what cannot be changed; interacting calmly and patiently; showing comprehension and empathy; adapting to changing needs, capacities and resources; and initiating shared activities. The strategies (3.5) and changes (4.5) regarding relationship quality have been explored in phase one of the study and are described in detail in the SRQD model [24].

## Supportive intervention components (level 1 in Fig. 1)

Caregivers mentioned five programme components which were helpful to develop and apply effective coping strategies in their daily lives (intervention components 1.1 – 1.5). Regarding the didactic strategies to provide information and training and facilitate knowledge transfer, caregivers most frequently described the active skills training in the group as essential, followed by sharing experiences and knowledge in the group. The five intervention components and the relevant didactic methods are described in the context of the supportive strategies (level 3) to illustrate which and how intervention components or didactic methods helped caregivers to develop and apply supportive strategies.

## Strategies applied by caregivers to achieve changes (level 3 in Fig. 1)

### Strategy: Finding ways to cope with problems in daily life (3.3)

During and after participation in the LFBHB intervention, caregivers increasingly reported situations in which they started to reflect on challenging situations related to caregiving. Reflecting on interactions, needs and perceptions regarding themselves and the person living with dementia increased their understanding and knowledge of behaviours precipitating negative reactions in both members of the caregiving dyad, such as dismissive or aggressive behaviour. It also helped them to better understand behaviours leading to positive reactions in both dyad members, such as motivation and interest in participating in activities. The systematic procedure learnt in the LFBHB intervention guided caregivers to first reflect on a challenging situation, identify thoughts and related emotions, differentiate between modifiable and unmodifiable aspects and then select appropriate strategies.

*‘The systematic [procedure], how to order facts and emotions. [..] This had an enormous impact on me and helped me advance the most. I can use this again and again. What does it do to me, how do I respond to it? I can now work on that because I have a framework. That helped me the most’ (Spouse CG2, t2).*

Differentiating between modifiable and non-modifiable aspects was described as an essential step of the systematic procedure and a strategy for caregivers to identify when they could search for and apply practical solutions or when they had to change their thinking and perspective (reframing).

*‘We know better where to act. We can accept things better, when he [parent with dementia] shows us that something is unchangeable for him. Differentiating between modifiable and non-modifiable was an essential aspect which helps us in our daily life’ (Adult child CG5, t1).*

For non-modifiable aspects, during and after the intervention, caregivers provided increasingly more examples of how they changed their perspective to mitigate their own painful and burdensome emotions or those of their family member living with dementia. Caregivers frequently explained how they learned to let go of concerns that cannot be remedied; for example, they did so by worrying less about the future or not striving to change dementia-related behaviours but instead changing their expectations. They further provided examples of how they endeavoured to view matters more positively, such as focusing on the things which still existed or worked rather than on losses or deficits.

*‘It was very new for me, that we can differentiate between thoughts and emotions and classify them, and this can bring calm. Also, the aspect of distorting views [cognitive distortions], that we could see everything differently and have more positive thoughts’ (Spouse CG1, t1).*

During and after the LFBHB intervention, caregivers provided examples of how they increasingly searched for, applied and evaluated different practical solutions and thereby practiced using new strategies in their daily life. Caregivers reported employing the systematic procedure of problem-solving in their daily life to clarify their thoughts and emotions and to gain a clearer picture of possible and suitable solutions to address a stressful situation.

*‘The course helped me to assess the whole thing. It helped me to become calmer, to order things and to immediately apply the procedure in my head to reflect on the reactions. […] Compared to before, I rather see solutions than problems’ (Spouse CG3, t2).*

Caregivers attributed their success in applying the systematic procedure in daily life to the active skills training provided in each intervention session, wherein the procedure was applied to stressful situations under the guidance of the group leader and with the active participation of other caregivers in the group. They also benefited from discussing their experiences of applying the procedure and the related strategies in daily life in the group sessions, to receive further guidance for using them more effectively. Caregivers highlighted that they needed substantial guidance and practical training in the group and in their daily life before being able to successfully and automatically employ the procedure and the strategies by themselves. This was particularly the case for reframing.

*‘It took 14 days to three weeks before I started to think for myself. […] This framework [active skills training] was most helpful to get the necessary tools. There I was accompanied and guided. […] These few sessions provided me the basis to process and to use it in daily life’ (Spouse CG2, t2).*

Identifying dementia symptoms and the consequences of the disease in relation to the behaviours and needs of the family member living with dementia was essential for caregivers to recognise that their relative’s behaviour was not directed towards them and to better capture the needs of their family member. Specific knowledge about dementia and about caring for and communicating with a person living with dementia helped them to reflect on challenging situations and find adapted solutions. This knowledge further helped them to adapt their expectations, their behaviour and communication, their activities and the environment to reduce stressful situations. The most important didactic method in this regard was the active skills training, including the analysis of practical situations in the group and role plays, and the systematic procedure, prompting caregivers to transfer dementia-specific knowledge to their individual situation and to be creative in finding possible solutions.

### Strategy: Sharing experiences and knowledge (3.4)

The caregivers appreciated talking about their situation in small groups, with people who had similar experiences or with the group leaders. The small groups provided a safe environment where caregivers felt confident talking about their situation and negative feelings without inhibitions or feelings of guilt and where they received support to find solutions.

*‘I cried so much in the first meeting. This surprised me because at home, I never cry. It was a place [LFBHB group] where I could bring up the topic for the first time, where I had peers and real professionals. It made me feel good’ (Spouse CG20, t2).*

However, talking about one’s own experiences was sometimes challenging for caregivers, as it involved addressing painful aspects of their situation. This was particularly difficult for caregivers struggling to acknowledge their role or the losses they faced.

Sharing experiences also allowed caregivers to learn from each other by hearing how others solved certain problems or what could be important in later stages of dementia. This substantially broadened their representations of supportive strategies and the different consequences of dementia for the people affected and the different types of caregivers, such as spouses or adult child caregivers.

*‘We all have the same problems at home. We continued to discuss during the breaks. One said I coped with it this way and another said, it did it like this. Then I thought, this is good, I am going to try this as well’ (Spouse CG18, t2).*

During the active skills training to apply the systematic procedure, caregivers enjoyed having the opportunity to become actively involved by sharing their ideas and experiences, as it allowed them to receive acknowledgment for their competencies. They further appreciated the flexibility of the group leaders to adapt and provide relevant knowledge for the different situations discussed in the group sessions.

*‘I was glad, that we discussed one of my topics. I thought, there is no solution for this problem, but then with the brainstorming, I realised that I can actually change something. That helped me a lot’ (Spouse CG20, t2).*

Some caregivers shared their experiences and knowledge not only in the group but also with other family members during and after the intervention. Sharing knowledge and experiences with other family members promoted a shared understanding, facilitated communication and collaboration within the family and empowered other family members to participate more in care.

### Strategy: Finding yourself again (3.2)

Caregivers employed specific strategies to learn to focus on themselves again. They provided examples of reflecting on their own needs and responsibilities and on the importance and necessity of taking breaks and caring for themselves to remain well. Not all caregivers had taken actions in this direction until the last interview, but the majority expressed reflections and plans in this direction. Caregivers started with activities eliciting minimal resistance or requiring no outside support, such as dedicating a room in the house to time alone or planning regular short breaks in their daily routine. Caregivers highlighted that it took them time to introduce these new routines, as they had to relearn how to take time for themselves and sometimes needed to find feasible activities that brought them happiness. Some caregivers already took time off regularly before the intervention and continued doing this during and after the intervention.

*‘In the beginning, I put everything on hold, tennis and so on. Now, I go skiing again for example. This does me good. I realised he [partner with dementia] can […] be alone for two to three hours, no problem. And I am not worried. My children also tell me that I have to do my own things. Since I do this, I feel better’ (Spouse CG18, t2).*

Already before the intervention, caregivers expressed reflections and concerns about their limitations, such as how long they could continue without risking a burnout. During and after the intervention, they started reflecting more concretely about the responsibilities they could manage to bear, the amount and kind of care they could provide, as well as how they could balance the care they were providing with the extent of their own resources to remain well. They also communicated more about their needs, especially regarding respite, with the person living with dementia and family members.

*‘In the course we learned that we have to take breaks more often. If it is stressful for me to be with him all day, I will probably get ill one day. I want to avoid this. That is why I tried to take an hour or half an hour for myself a number of times’ (Spouse CG1, t1).*

Caregivers strongly valued the fact that the group leaders, as professionals, explained the importance of taking time for themselves and encouraged them to do so without feeling guilty. Caregivers were further encouraged by their counterparts to share their own practical methods of implementing breaks in daily life during the group sessions.

### Strategy: Seeking support (3.1)

Barriers to asking for help were a common theme before, during and after the intervention. Caregivers were sometimes reluctant to ask for support and involve others, for many reasons, such as fearing that the family member with dementia would not accept or appreciate the support. Other reasons included the following: feeling guilty for pushing away the person with dementia; and uncertainty about the appropriate time to incorporate support, the proper form of support and where to receive the support. This caused caregivers to avoid these issues experienced as uncomfortable and difficult to implement. During and after the intervention, caregivers expressed reflecting about their needs for support but also about the courage required to become active in order to receive and actually accept offers of support.

*‘The question is, how can I implement it? Do I have the courage to do this? […] How can I bring myself to say: now it is the time to do it? It is now my goal to do this in [next month]. To visit the day care centre and talk to the nurse there’ (Spouse CG9, t1).*

Becoming more aware of the importance of caring for oneself and realising they did not have to do everything by themselves encouraged caregivers to reflect on their needs and possibilities for support. Mainly after the intervention, caregivers involved family members or friends by informing them about their situation, asking for practical support, discussing responsibilities and tasks for different family members, or accepting their offers of support. Some caregivers took the first steps towards receiving formal support, such as by applying for financial support or organising admission to a day care centre.

*‘At the end, we saw all the support possibilities that actually exist. I have probably been muddling along for too long. I saw the support services and that is why I started to organise things for financial support. And now things are moving forward, a woman came for the financial support and next week another is coming from a support association’ (Spouse CG10, t2).*

Knowing what one needs and where to receive the corresponding support was described as an essential prerequisite to asking for support. The practical exercises where caregivers were guided to establish a list with support persons and to identify their concrete needs and preferred support options were described as empowering and motivating first steps towards reflecting on and communicating their needs. Practical information about formal support services was described as useful to reflect on and select services. Role plays were also found useful to practice communicating one’s needs for support or to make requests to family members or to the persons living with dementia.

*‘I think the solution in the coursebook is very good, that we have to deal with this [seeking support] at an early stage and not wait and put off the problem. We also practiced, what needs do I have? Who could I ask for support and how? Who is in my support network? […] I have already spoken to two people, and to my children, so we can discuss this all together’ (Spouse CG6, t1).*

## Facilitators and barriers (level 2 in Fig. 1)

The facilitators and barriers describe contextual aspects related to caregiver factors which facilitated or prevented caregivers from developing and applying supportive strategies and illustrate the fact that caregivers differed in terms of the changes they achieved regarding calmness. The main barriers were limited physical resources (e.g. due to health problems) or limited emotional resources to accept losses due to dementia or the caregiver role. Having a poor relationship before the onset of dementia was another barrier described by the caregivers. Conversely, the learning process of the caregivers was facilitated by the ability to accept to some extent both the disease and the caregiving role, the presence of a positive relationship before the onset of dementia and a positive attitude of the caregiver. ‘Experiencing calmness’ was found to positively influence certain barriers or to further promote facilitators. For example, when feeling calmer, caregivers reported having more cognitive and emotional resources to reflect, find solutions and cope with losses. As the facilitators and barriers in the calmness and SRQD models overlap with each other, we refer the reader to the more detailed description provided in the publication on the SRQD model [24].

## Changes between pre- and post-intervention scores

Quantitative outcomes (see Table 4) indicated a significant decrease in caregivers’ psychological distress and distress related to the behavioural problems of the person living with dementia, as well as a significant increase in caregivers’ self-efficacy. The decrease in the subjective burden did not reach statistical significance (small effect size).

**Table 4:** Changes in quantitative outcomes between the pre and post-test

	Pre-test (N=22) Md (Q1 – Q3)	Post-test (N=22) Md (Q1 – Q3)	Wilcoxon Z (df=21)	p-value (one-tailed)	Effect size (d)
Burden (0-88)	26.50 (17.50 – 39.50)	25.25 (18.75 – 35.50)	1.01	0.157	0.19
MBP (0-4)	1.29 (1.04 – 1.91)	1.35 (1.12 – 1.90)	0.89	0.188	-0.05
MBP-related distress (0-4)	1.74 (1.47 – 2.20)	1.46 (1.22 – 1.95)	1.69*	0.045	0.44
Psychological distress (14-56)	25.00 (20.50 – 31.00)	22.00 (17.75 – 26.00)	1.90*	0.029	0.45
Self-efficacy (0-10)	7.00 (5.50 – 8.00)	7.25 (7.00 – 9.00)	1.75*	0.041	-0.48

For all the variables listed, higher scores indicate higher levels; Md: Median; Q1: first quartile; Q3: third quartile; MBP: memory and behavioural problems;

^*^p<.05 (one-tailed); for burden, scores above 18 indicate a heavy burden, and scores above 32 indicate a severe burden

## Discussion

The current study allowed us to explore changes and related processes in the context of the LFBHB intervention, following its shortening and adaptation to the different cultural context in Switzerland. The resulting calmness model adds to our understanding of how this intervention works by identifying its key intervention components, mechanisms of change and contextual aspects facilitating or preventing changes in caregivers. Such knowledge is highly relevant to help further develop, evaluate and successfully implement a complex intervention and, more specifically, to refine the intervention’s programme theory and to optimise the intervention [19–22]. Knowledge about the intervention’s key components is also useful for health care professionals and researchers aiming to develop other interventions targeting similar mechanisms or aiming to achieve similar changes [20–22]. In addition, knowledge about the facilitators and barriers to change may help to identify which caregivers can benefit the most from the intervention and which caregivers may need a different type of support [20, 37].

### Calmness as the main change for caregivers, triggered by multifaced interlinked mechanisms

The approach used has allowed us to explore key changes and related mechanisms from the perspective of the caregivers, revealing that experiencing calmness was a core change for them in addition to relationship quality, both being interrelated. The calmness model suggests that this new core outcome is facilitated by five associated or intermediate changes, such as having time for oneself and being able to handle daily life. Of these five intermediate changes, having positive interactions highlights the role of relationship quality at different levels of the change process. Such intermediate changes or outcomes provide relevant information about how the intervention’s key components function and regarding relevant processes, intervention mediators and contextual factors [38]. An important finding of our study is that calmness shares a bidirectional link with these intermediate changes and that the latter interacted and reinforced each other, all being facilitated by the same intervention components. This illustrates that changes in the LFBHB intervention occur through a multifaceted process involving interlinked changes and mechanisms of change. This suggests that an intervention might not need to directly target all the potentially beneficial changes, as doing so might unnecessarily complexify the intervention content and require excessive intervention sessions. Only the intervention components identified as most effective could be used to avoid overburdening caregivers [39].

### The role of intermediate changes and related mechanisms

Findings regarding the core change ‘experiencing calmness’, the intermediate changes and related mechanisms provide hypotheses about the mechanisms leading to the changes observed in the quantitative intervention outcomes. For instance, increased self-efficacy is very similar to the intermediate change ‘being able to handle daily life’, which largely contributed to experiencing calmness. Indeed, knowing how to positively change a stressful situation and having had successful experiences in this regard is known to be essential for increasing perceived control over the situation and trust in one’s ability to change the situation [40]. Reduced psychological distress in caregivers could be favoured by the sharing of experiences in the LFBHB group, which validated caregivers’ experiences and emotions, as well as creating a sense of relief [17, 18] and reducing feelings of isolation. Reduced psychological distress could also be achieved through experiencing positive interactions with their family member with dementia, which includes less dysfunctional interactions and helps caregivers to feel less alone. The intermediate changes provide some evidence, in line with the literature review by Wiegelmann et al. [41], that our multi-modal psychoeducational intervention was able to increase caregivers' calmness, in other terms to decrease their stress level, by improving a broad range of interrelated factors. These factors include coping, social support, self-confidence and the caregivers’ subjective perceptions of their situation. However, the role of intermediate changes and their relationship with the quantitative intervention outcomes would need to be quantitatively tested in a larger sample. The calmness model provides a basis to identify outcomes which should be tested in this next step.

### Reframing as a key component

Reframing was found to be a key component amongst all strategies applied by caregivers to experience calmness, as it facilitated all intermediate changes. The importance of reframing and its uses as described in this study largely correspond to the process evaluation findings in Canada, where reframing was also found to be the most useful strategy and was applied by most caregivers [16]. Caregivers in our study applied reframing in diverse ways to reduce their own or their relatives’ painful and burdensome emotions. This included, for example, focusing on positive aspects of the caregiving situation or relinquishing inappropriate control and excessive responsibility. Indeed, according to Cheng et al.’s review [39] concerning psychological ingredients of dementia caregiver interventions, self-efficacy in controlling upsetting thoughts is a more important predictor of caregiver outcomes than self-efficacy in managing challenging behaviours or receiving support and is thus a ‘primary mechanism of change in psychological interventions based on the cognitive model’ [39, p.2]. In a Cochrane review [42] including 11 RCTs, cognitive reframing was found to be a useful tool to individually support family dementia caregivers, as it can be adapted to different personal situations and applied to various problems.

Problem-solving was reported as helpful and important in both Switzerland and Canada, although it was the least commonly mentioned coping strategy in Canada [16–18]. This was not the case in Switzerland, where it was the second most frequently used after reframing and where caregivers provided numerous practical examples of how they used problem-solving in their daily lives. In both contexts, problem-solving was often intermingled with reframing, as caregivers used both strategies simultaneously. Thus, caregivers may not have been fully aware of which strategy they were using. The analysis of the longitudinal qualitative data (phase 1) revealed that caregivers were more aware of using the different strategies and provided more concrete examples in the interviews conducted during the intervention, compared to the interviews at the end of the intervention. This suggests that caregivers may have already integrated these strategies before the end of the intervention and may have been less aware of using them [24, 25]. This might also have been the case in Canada, where interviews for the process evaluation were only performed before and after the intervention.

Actions to seek support were less frequently mentioned and were mainly taken when they were in the power of the caregivers rather than depending on other people, whereas the importance of seeking support was more present in the reflections of the caregivers. In Canada, only 12 of the 30 participants referred to seeking support [16]. Lavoie et al. [16] argued that caregivers may not have sufficient control over the decision to involve support. Although caregivers can develop the relevant skills and knowledge regarding support seeking, external aspects can prevent them from doing so, such as the reluctance of the person living with dementia or disagreements in the family regarding the support needed. Another reason for the limited implementation of actions to seek support might be the late introduction of this topic. Seeking support was the last strategy presented in both the long and short versions of the intervention. Hence, caregivers had less time to implement these actions until the post-intervention interview. Indeed, seeking support can involve several time-consuming steps, such as finding and asking the right persons; empowering them and organising shared activities; and identifying, organising and attempting different support services before finding the right one.

### Active skills training in the group as the most empowering didactic method

Working on practical situations in the group was the most important for caregivers to learn to apply the systematic procedure and the coping strategies independently; to enhance their reflective skills; and to transfer knowledge and skills to their personal situation. Applying this method in the group allowed all caregivers to participate actively, which is known to be beneficial for the learning process [43, 44]. Moreover, caregivers learned from each other, especially when witnessing how others managed to apply effective coping strategies and how they benefited from applying these strategies. This type of vicarious experience is described in the literature as a source of self-efficacy [45]. Bandura [45] describes role models, who also experience difficulties and need to find means to cope with them, as being particularly effective, as they can increase the confidence in the observers and demonstrate useful strategies to cope with certain difficulties [45, 46].

However, the most effective source of self-efficacy is when people successfully performed a task by themselves (mastery experience) [45]. This can be promoted by providing opportunities to experience new behaviours (e.g. in the form of classes where participants are trained to gain and apply the necessary skills) [46]. Many such opportunities were provided in the LFBHB intervention, where the health care professionals closely guided and trained the caregivers to reflect on their situation, and to choose and apply appropriate coping. They thereby supported caregivers in successfully applying the learnt procedures and perceive the benefits resulting from this effort. This guidance included practical exercises in the group to work on individual problems, role plays and structured exercises at home with subsequent debriefing in the group. Experiences of success when performing a task and self-efficacy can then reinforce each other and can act as an upward spiral [46, 47]. In this line of thought, self-efficacy has been described as a key factor not only to predict and explain the successful adoption of behaviour changes but also to maintain them [46].

The benefits of the group format were highlighted in the process analysis in Canada [16] as well as in all studies evaluating the LFBHB intervention in Switzerland [17, 18]. Caregivers in both contexts appreciated having a safe environment to discuss their stressful experiences and emotions as well as the possibility to learn from each other and receive acknowledgment for their experiences and knowledge. This benefit is typical of support groups [43, 44].

### Future research

Our qualitative exploration resulted in a model summarising the multifaceted relevant changes in the caregivers and the related mechanisms which make the intervention work. These findings expand upon the quantitative results of previous studies by shedding light on the change mechanisms of this intervention. As this knowledge base is purely qualitative and relies on a small sample, it would require a subsequent step of quantitative testing on a larger scale.

Identified barriers, such as difficulties in accepting losses and the caregiving role or limited resources, prevented some caregivers from developing and applying new knowledge and skills [24]. Such barriers suggest a possible need for a selection of participants or of better timing for the participation, which should be further explored in the context of the LFBHB intervention, or of other psychoeducational interventions. Particularly for such interventions requiring substantial involvement from the caregivers and non-neglectable health costs, it is essential to better define which caregivers can benefit from the intervention, while others may need a different kind of support and/or should delay participation. In addition, the barriers and facilitators described in our model focus on factors related to the participants. Future studies should explore other contextual aspects such as the skills of a group leader or the composition of the groups. Regarding the latter, for example, exploring differences between groups performed with spouse caregivers only or with mixed groups, including both adult child and spouse caregivers, could yield valuable insights.

### Limitations

The sample primarily included women and spouse caregivers, indicating that aspects that are more relevant for male or adult child caregivers might not be sufficiently illustrated in the findings. This might also be the case for caregivers of people with particularly challenging types of dementia, such as frontotemporal dementia, which was the case for only two caregivers in our sample. The quantitative outcomes are based on a small sample of 22 caregivers. However, the results are highly similar to the changes found among the 47 Swiss French-speaking caregivers in our second feasibility and pilot study [18]. Another limitation is related to the fact that the calmness model was developed in two phases, the first focusing on relationship quality and the second on all other changes in the context of the LFBHB intervention. Even though the changes identified in the caregivers were strongly interlinked between the two phases, some aspects might have been overlooked due to this sequential approach.

## Conclusion

The calmness model extends the understanding of how the LFBHB intervention works by identifying key change processes and mechanisms from the caregivers’ perspective. Reframing was the most important key component, as was also observed in other interventions. Our study suggests that this importance is related to caregivers being able to use this coping strategy in very diverse ways, which in turn positively impact a range of outcomes. Training in practical skills with the active participation of the caregivers under the close guidance of a professional group leader was the most powerful didactic method in helping caregivers to acquire, transfer and apply new knowledge and skills in their daily lives.

## Supplementary Information

Additional file 1. LFBHB intervention components and associated didactic tools used to provide information and training.

Additional file 2. Post-intervention interview guide.

Additional file 3. Psychometric measures of measurement instruments.

## Data Availability

The datasets used and/or analysed during the current study are available from the corresponding author on reasonable request.

## Abbreviations

CG: Caregiver
LFBHB: Learning to feel better, to help better
PlwD: Person living with dementia
RCT: Randomised controlled trial
