# Supplementary material for: A qualitative exploration of changes and mechanisms of changes in a psychoeducational intervention for family dementia caregivers
Source: BMC Prim Care. 2024 Sep 28;25:353. doi: 10.1186/s12875-024-02602-2 (PMC11437664; doi:10.1186/s12875-024-02602-2)
Supplement: Supplementary file 1 — Additional file 1. LFBHB intervention components and associated didactic tools used to provide information and training. [file 12875_2024_2602_MOESM1_ESM.pdf]

Additional File 1. LFBHB intervention components and associated didactic tools used to provide information and training

**Global procedure for the appraisal of stressful situations and coping** (Based on the transactional theory of stress and the coping and intervention framework by Folkman et al. [1])	
**Intervention Components**	**Didactic tools for information provision, training and knowledge transfer**
**1) Appraisal of stressful situations**	
Analysing the situation, shifting from global stressors to specific stressors (what is stressful in a situation and which are the emotional reactions)<br>Identifying what can be changed and what cannot be changed in a situation<br>Choosing an appropriate strategy to manage the situation (problem solving, reframing for unmodifiable situations, seeking for social support)	**Course book:** scheme, different steps, working sheets on how to use the global procedure<br>**Educational video:** global procedure for stress management with explanations and practical examples<br>**Practical exercises in the group:**<br>1) Specifying currently difficult situations, identifying modifiable or unmodifiable situations<br>2) Stress management procedure: applying all relevant aspects of the stress management procedure in the participants' current stressful situations<br>**Sharing experiences in the group:** current stressful situations
**2) Coping strategies** - Choosing an appropriate strategy depending on whether the situation can be modified or not	
**2.1) Problem-solving techniques** for modifiable situations (seven-step procedure)	**Course book:** theoretical knowledge, practical examples and working sheets on problem solving<br>**Educational video:** problem solving with explanations and practical examples<br>**Practical exercises in the group:**<br>1) Applying problem solving to the participants' modifiable stress situations<br>2) Practicing problem solving
**Effects of dementia on behaviour and relationships, approach and communication techniques**<br><br>Functions of short and long-term memory<br>Memory disorders and the role of the emotional memory<br>Handling cognitive disorders<br>Communication	**Course book:** theoretical knowledge and practical examples on:<br>1) Dementia-related knowledge about consequences of the disease on capacities and behaviour of person living with dementia<br>2) Communication and care approaches to prevent tensions<br>**Educational video:** effects of cognitive disorders on behaviour and relationships and communication approach and techniques, providing theoretical knowledge and practical examples<br>**Sharing experiences in the group:** difficult experiences regarding communication and strategies applied in these situations<br>**Role-playing exercise:** situation where communication is difficult (identifying strategies which are not optimal, trying new strategies)<br>**Exercises at home:**<br>1) Reflecting about a specific situation where communication with the person living with dementia is difficult<br>2) Practicing new approach/communication techniques
**2.2) Reframing** for unmodifiable situations (looking at things from another angle to reduce painful emotions)<br><br>Common problems related to dementia that cannot be changed<br>Recognising dysfunctional behaviours as dementia-related rather than deliberate behaviours<br>Relations between thoughts and emotions<br>Reframing unhelpful thoughts, changing perspectives<br>Identifying unhelpful thoughts	**Educational videos:**<br>1) Reframing, relations between thoughts and emotions, recognising distorted thoughts<br>2) Reframing, knowing and accepting the disease, acknowledging grief, recognizing one's own limits and appreciating the rewarding aspects of the caregiver role<br>**Course book:** theoretical knowledge, practical examples and working sheets related to reframing<br>**Exercises in the group:**<br>1) Illustrating the relations between situation-thoughts-emotions, showing how less distorted thoughts changes

Concentrating on present reality, looking at the person living with dementia with his or her present abilities, accepting daily losses<br>Recognising one's own limits, appreciating rewarding aspects of the caregiver role	2) Reminder of the relations between situation-thoughts-emotions, identifying thoughts in emotionally painful situations<br>3) Connecting the theory to one's own situations, applying reframing to participants' unmodifiable difficult stress situations<br>4) Countering stress by "treating yourself" (sharing/exploring ideas), accepting and communicating one's limits<br>*5)* Relaxation or mindfulness exercises<br>**Exercises at home:**<br>1) Applying reframing and countering stress by "treating oneself"<br>2) Identifying personal support networks<br>**Sharing experiences**: importance of caregiver activities, responsibilities, caregiver contributions, valuing rewarding aspects
**2.3) Support seeking** (for modifiable or unmodifiable situations)<br>Seeking support from personal networks, professionals or volunteers; differences between formal and informal forms of support<br>Reluctance to ask for support, applying reframing to these thoughts<br>Identifying support networks<br>Type of informal/formal support (which type of support is needed; who can optimally provide it)<br>Identifying own support needs<br>How to ask for support<br>Maintaining received support	**Course book and practical information by the group leader**: theoretical knowledge, practical examples and working sheets related to formal support services<br>**Educational video:** seeking support (importance of asking for the right person the right type of support, differences formal-informal, how to ask for support, reluctance to ask for support)<br>**Practical exercises:**<br>1) Identifying and establishing support network<br>2) Specifying clear and achievable requests, identifying reluctances<br>3) Planning a request for support (type, target person, what-when-how-where, barriers and facilitators) and formulating the action plan<br>**Role-play exercise:** asking for support<br>**Exercise at home:** identifying known formal support (services), make a request for informal support

Note. LFBHB: Learning to feel better… and help better, Table content based on Lévesque et al. [2], extended and adapted for the shortened Swiss Version of the LFBHB intervention. More details about the Swiss version of the intervention can be found in Pihet et al. [3]. The intervention content and methods developed and used in the context of the original intervention in Canada are described in detail in Lévesque et al. [2].

References

1. Folkman S, Chesney M, McKusick L, Ironson G, Johnson DS, Coates TJ. Translating Coping Theory into an Intervention. In: Eckenrode J, editor. The Social Context of Coping. Boston, MA: Springer US; 1991. p. 239-60.
2. Lévesque L, Gendron C, Vézina J, Hébert R, Ducharme F, Lavoie JP, et al. The process of a group intervention for caregivers of demented persons living at home: conceptual framework, components, and characteristics. Aging Ment Health. 2002;6(3):239-47. https://doi.org/10.1080/13607860220142468
3. Pihet S, Clément M, Terrapon E, Kipfer S. Adaptation of a psycho-educational group programme to improve coping in dementia caregiving: a feasibility study with mixed-methods. BMC Geriatrics. 2024;24(1):197. https://doi.org/https://doi.org/10.1186/s12877-024-04815-7
