# Supplementary material for: A qualitative exploration of changes and mechanisms of changes in a psychoeducational intervention for family dementia caregivers
Source: BMC Prim Care. 2024 Sep 28;25:353. doi: 10.1186/s12875-024-02602-2 (PMC11437664; doi:10.1186/s12875-024-02602-2)
Supplement: Supplementary file 2 — Additional file 2. Post-intervention interview guide. [file 12875_2024_2602_MOESM2_ESM.pdf]

Additional File 2. Post-intervention interview guide

Evaluation of the "Learning to feel better… and help better" programme

Please note that questions could be adapted or added to deeper explore themes raised by the participants during the interviews.

**First open questions**	**Notes**
Could you tell me about your experiences of taking part in the programme?<br>Could you tell me how you feel about taking part in the programme?<br><br>*Examples of follow-up questions in relation to themes raised by participants:*<br>• *Exploring how they experienced the themes they have just described, e.g.*<br>*How do you experience the change(s) you have described? (refer to the change(s) described by the participant)*<br>*Could you tell me more about how you handle such situations in your daily life as a caregiver?*<br>*How did you feel about sharing experiences in a group with other caregivers?*<br>• *Exploring related processes, e.g.*<br>*What, do you think, contributed to this change you just described? (refer to the change described by the participant)*<br>*What, do you think, helped you to handle the situation in this way?*<br>*Could you tell me more about any challenges you experienced while trying to apply this strategy? (refer to the strategy/action(s) described by the participant)*<br><br>**Intermediate questions**<br>• When you took part in the programme, what did you appreciate most?<br>• What were positive aspects for you when taking part in the programme?<br>• Could you tell me what you particularly appreciated about this/these positive aspect(s)? (refer to the positive aspect(s) raised by the participant)	

• When participating in the programme, what were the most negative aspects for you?<br>• Were there any things that were difficult for you, and if so, could you tell me more about them?<br>• Could you tell me more about how you experienced this/these negative or difficult aspect(s)? (refer to the negative/difficult aspect(s) raised by the participant)?<br><br>• How do you feel about the programme in relation to the expectations you had before you started?<br>• Have there been any surprises for you?<br><br>• Are there any things about the programme that you would like to see changed or improved?<br>• When you took part in the programme, were there any other things you would have needed?<br><br>**Course material**<br><br>Could you tell me about your use of the course material (educational videos, course book)?<br><br>*Examples of follow-up questions:*<br><br>• *Could you tell me more about whether and how you used the course book / the educational videos at home?*<br>• *Was there anything you liked about the course book / the educational videos?*<br>• *Did you find any negative points in the course book / the educational videos?*<br>• *Are there any topics you would have liked to know more about?*<br>• *Is there any other support material or information that would have been useful for you to have as a caregiver?*<br><br>**Organisation of the programme**<br><br>What do you think of the organisation of the course (3-hour sessions, breaks, time and place of the course)?	

**Ending questions**<br><br>Is there anything else I need to know to help me better understand what you have experienced while participating in the programme?<br><br>Is there anything important that you would like to talk about or that we have not talked about?<br><br>Would you like to add something?	
