# Supplementary material for: A qualitative exploration of changes and mechanisms of changes in a psychoeducational intervention for family dementia caregivers
Source: BMC Prim Care. 2024 Sep 28;25:353. doi: 10.1186/s12875-024-02602-2 (PMC11437664; doi:10.1186/s12875-024-02602-2)
Supplement: Supplementary file 3 — Additional file 3. Psychometric measures of measurement instruments. [file 12875_2024_2602_MOESM3_ESM.pdf]

Additional File 3. Psychometric measures of measurement instruments

Quantitative outcome	Measurement instrument	Psychometrics measures
Caregivers' burden	Zarit Burden Interview [1, 2]	The German validation showed high internal consistency (Cronbach's α = 0.92) and strong correlations with caregivers' life satisfaction and depression scores, as well as with the dependency scores of persons with dementia, dementia severity, the severity of neuropsychiatric symptoms and caregivers' distress due to these neuropsychiatric symptoms [2].
Memory and behavioural problems (MBP) and caregivers' MBP-related distress	Revised Memory and Behavior Problems Checklist (RMBPC) [3]	The original English version has good overall scale reliability, with alphas of 0.84 for the behaviour of persons with dementia and 0.90 for caregivers' reactions; confirmed validity through correlations of RMBPC scores for depression and cognitive impairment amongst persons with dementia, as well as caregivers' depression and stress.<br>The German version used in the study was forward and backward translated with a standardised method for use in a randomised controlled trial with 92 informal dementia caregivers [4]; no psychometric validation in German had been found at the time of data collection.
Caregivers' psychological distress	Ilfeld Psychiatric Symptoms Index – short version [5]	The original English version showed satisfactory internal consistency (Cronbach's α = 0.91); subscales identified by factor analysis; and concurrent validity demonstrated against three relevant criteria [5].<br>The questionnaire was forward and backward translated into German for use in the study, as no translation or psychometric validation in German had been found at the time of data collection.
Caregivers' self-efficacy	Bandura [6]	The item about confidence regarding the ability to assume the caregiver role was forward and backward translated into German.

Note. MBP: memory and behavioural problems, RMBPC: revised memory and behavior problems checklist

References

1. Zarit S, Orr N, Zarit J. The hidden victims of Alzheimer's disease: Families under stress. New York: New York University Press; 1985.
2. Braun M, Scholz U, Hornung R, Martin M. The burden of spousal caregiving: a preliminary psychometric evaluation of the German version of the Zarit burden interview. Aging Ment Health. 2010;14(2):159-67. https://doi.org/10.1080/13607860802459781
3. Teri L, Truax P, Logsdon R, Uomoto J, Zarit S, Vitaliano PP. Assessment of behavioral problems in dementia: the revised memory and behavior problems checklist. Psychology and aging. 1992;7(4):622-31. https://doi.org/https://doi.org/10.1037//0882-7974.7.4.622
4. Berwig M, Heinrich S, Spahlholz J, Hallensleben N, Brähler E, Gertz H-J. Individualized support for informal caregivers of people with dementia - effectiveness of the German adaptation of REACH II. BMC geriatrics. 2017;17(1):286-. https://doi.org/10.1186/s12877-017-0678-y
5. Ilfeld FW. Further validation of a psychiatric symptom index in a normal population. Psychological Reports. 1976;39(3, Pt 2):1215-28. https://doi.org/https://doi.org/10.2466/pr0.1976.39.3f.1215
6. Bandura A. Self-efficacy: toward a unifying theory of behavioral change. Psychol Rev. 1977;84(2):191-215. https://doi.org/https://doi.org/10.1037//0033-295x.84.2.191
